# Soret separation and thermo-osmosis in porous media

**DOI:** 10.1140/epje/s10189-022-00194-2

**Published:** 2022-05-03

**Authors:** Bjørn Hafskjold, Dick Bedeaux, Signe Kjelstrup, Øivind Wilhelmsen

**Affiliations:** PoreLab, Department of Chemistry, Norwegian University of Science and Technology - NTNU, 7491 Trondheim, Norway

## Abstract

**Abstract:**

When a temperature difference is applied over a porous medium soaked with a fluid mixture, two effects may be observed, a component separation (the Ludwig–Soret effect, thermodiffusion) and a pressure difference due to thermo-osmosis. In this work, we have studied both effects using non-equilibrium thermodynamics and molecular dynamics. We have derived expressions for the two characteristic parameters, the Soret coefficient and the thermo-osmotic coefficient in terms of phenomenological transport coefficients, and we show how they are related. Numerical values for these coefficients were obtained for a two-component fluid in a solid matrix where both fluid and solid are Lennard–Jones/spline particles. We found that both effects depend strongly on the porosity of the medium and weakly on the interactions between the fluid components and the matrix. The Soret coefficient depends strongly on whether the fluid is sampled from inside the porous medium or from bulk phases outside, which must be considered in experimental measurements using packed columns. If we use a methane/decane mixture in bulk as an example, our results for the Soret coefficient give that a temperature difference of 10 K will separate the mixture to about 49.5/50.5 and give no pressure difference. In a reservoir with 30% porosity, the separation will be 49.8/50.2, whereas the pressure difference will be about 15 bar. Thermo-osmotic pressures with this order or magnitude have been observed in frost-heave experiments.

**Graphic abstract:**

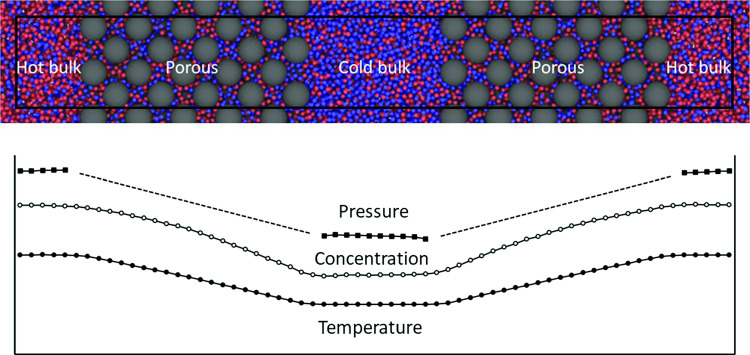

## Introduction

Thermodiffusion, the Ludwig–Soret effect, is the process by which a temperature gradient in a fluid or solid drives mass diffusion in a binary or multicomponent mixture. The effect bears the name of the scientists who discovered it, Carl Ludwig in 1865 and Charles Soret in 1879 [1, 2]. Research on thermodiffusion gained momentum with the Manhattan project, where Clusius-Dickel columns [3] were used to enrich uranium. Over more than 50 years, the effect has been extensively studied both experimentally and by computer simulations; a good review was given by Köhler and Morozov [4]. Many models have been developed to describe and explain the effect, based on kinetic theory [5], equilibrium and non-equilibrium thermodynamics [6], and computer simulations [7, 8], but a complete theory is not yet available, not even for bulk fluids.

Thermodiffusion in porous media is both challenging and interesting. Challenging, because the presence of a solid matrix adds complexity compared with bulk fluids due to the irregular pore structure and the fluid–matrix interactions [9, 10]. Interesting, because it concerns so many systems of natural and industrial importance. It has been speculated that thermodiffusion in hot subsea water plumes has played a role in the origin of life on Earth [11]. Three examples of thermodiffusion in porous media are: frost heave which damages roads at spring time [12], diffusive transport of reactants and products in heterogeneous catalysis [13], and formation and growth of salt lenses (columnar crystals) in the carbon cathode during aluminum electrolysis [14, 15]. Much attention has been given to the initial states of oil and gas reservoirs where the assessment of the composition of the hydrocarbon column may be seriously off if thermodiffusion is neglected [16–18]. Gravity segregation makes the heavier components in the hydrocarbon mixture migrate to the bottom of the reservoir, but Montel and coworkers [17] showed that thermodiffusion can counteract the segregation. The geothermal gradient makes the reservoir hot at the bottom, such that thermodiffusion may drive the lighter components to the bottom.

Classic experimental techniques for measuring the Soret effect involve porous media. Packed columns were from the early 1970s a dominant experimental apparatus for measuring Soret coefficients (see Ref. [9] and references therein). Moreover, packed columns resemble natural porous media, such as oil and gas reservoirs, and can therefore help understand why porous media behave differently from bulk fluids with respect to thermodiffusion. Costesèque and coworkers raised the question in publications from 2004 of whether the Soret coefficient is the same in a bulk fluid as in a porous medium [19, 20]. They used packed columns to measure the Soret coefficient and wanted to know if the porosity and tortuosity of the porous medium affected the measurements. They concluded that although the dynamics of mass separation was different, the measured coefficient in an aqueous copper sulfate solution was the same at steady state. In another study from 2010, Davarzani and coworkers showed the same for gas separation in porous media down to a porosity of about 28%. There were large effects on the separate diffusion and thermodiffusion coefficients, but no effect on the ratio between them, the thermodiffusion factor [21]. The effect of porosity was studied by Colombani and coworkers [22] using molecular dynamics simulations with a binary fluid mixture in a porous matrix. They did indeed show a small but significant effect of porosity down to about 70% porosity.

Extraction of data for the Soret effect from experiments involves coupled transport equations which are more complex than for bulk fluids. For instance, in a stagnant bulk fluid subject to a temperature gradient, the pressure equalizes in the system (neglecting gravity) due to the low resistance to fluid flow, but in a porous medium it does not [23]. A temperature gradient may drive an osmotic process, thermo-osmosis, which is typically found in membranes with a temperature difference over the membrane. Some papers on the Soret effect in porous media discuss this question indirectly or in part. Davarzani et al. used a volume-averaging technique to relate “effective” transport coefficients to the corresponding coefficient in a bulk fluid [24]. They found that in the diffusive regime, which is the regime of interest here, the effective Soret coefficient is equal to the bulk-fluid value. On the other hand, Faissat et al. used irreversible thermodynamics to derive a balance equation at stationary state (zero mass fluxes) which includes temperature- and composition gradients as well as gravity as driving forces [25]. They pointed out that “... the presence of a capillary plays an important role in the phenomenon, so that the characteristics of the porous medium must be taken into account in the description of real thermodiffusion.” Haugen and Firoozabadi [26] introduced a “pressure diffusion coefficient” in the mass flux, which multiplied with a pressure gradient, contributes along with Fickian and thermodiffusion to the total diffusive mass flux.

The effects of porosity were studied by Colombani et al. using a Lennard–Jones model for an equimolar methane-decane mixture with fixed “obstacles” and with interactions between the fluid and the obstacles [22]. They found that the Soret coefficient was lowered by about 30% at 75% porosity (compared with the bulk fluid) and that the reduction depended strongly on the structure of the porous medium. A pressure gradient was not a parameter in this study, probably because the porosity was not low enough to quantify the effect of thermo-osmosis, and gravity was not included.

To our knowledge, a study of a possible coupling between thermodiffusion and thermo-osmosis in a medium with two components and low porosity has not been made. The question is how the osmotic pressure affects the component separation. The effect of gravity, which gives a hydrostatic pressure gradient, was studied by Galliero and Montel [27], but because gravity acts directly on the masses of the fluid molecules, whereas thermo-osmosis does not, the hydrostatic pressure and the osmotic pressure have different relations to the Soret effect. It must therefore be expected that the origin of a pressure gradient, whether it is gravity or a temperature difference, will have different impacts on component separation and the Soret effect.

The purpose of the present work is to find what effect the porous medium has on the Soret coefficient and the coupling between thermodiffusion and thermo-osmosis. For this purpose, we have used non-equilibrium molecular dynamics to simulate a porous medium soaked with a two-component fluid mixture. This is a three-component system, but the porous matrix is frozen in the sense that matrix particles do not move. The fluid is an isotope mixture, a simple and common reference case, which has been well studied by computer simulations [7, 8]. When we now take this system to porous media, we will be able to separate between well-known trends reported in the literature, and properties particular of porous media. We find, for instance, the usual situation that the heavy particles migrate to the cold region. This simple mixture will also allow us to demonstrate the other characteristic property of the porous medium, the thermo-osmotic coefficient.

Our background and motivation for this work comes from studies of the Soret effect, which gives some emphasis on thermodiffusion. Important pioneering work on the other part of this work, thermo-osmosis, was done by Denbigh and Raumann [28, 29].

The theory presented in Sect. 2 starts with a summary of elements from nonequilibrium thermodynamics, viz. the entropy production due to the transport processes and the flux–force relations. This part builds on, and extends, work by Katchalsky and Curran [30], Førland et al. [31], and others. The results are general equations that relate the Soret and thermo-osmotic coefficients to the conductivities defined by the flux–force relations. The model system is introduced in Sect. 3, where the molecular dynamics simulations are described. We have used two system configurations, one with a completely space-filling porous medium, and the other with bulk fluid on both sides of the porous medium. Results for the Soret and thermo-osmotic effects are presented in Sect. 4 and discussed in Sect. 5. In particular, we discuss how these effects depend on the porosity of the medium and on the difference in wettability of the two fluid components.

## Coupled heat an mass transport in a porous medium

The entropy production for coupled heat- and mass transport in a two-component fluid in a porous medium without any other external forces than a temperature gradient may be expressed as:

1 $$\begin{aligned} \sigma = J_q^\prime \nabla \left( \frac{1}{T} \right) - \sum _{k=1}^2 J_k \frac{1}{T} \nabla \mu _{k,T} \end{aligned}$$

where $J_q^\prime$ is the measurable heat flux, $J_k$ is the molar flux of component *k*, *T* is the temperature, and $\mu _{k,T}$ is the sum of the pressure- and compositional contributions to the chemical potential (excluding the thermal). Components 1 and 2 are the miscible fluid components. The porous matrix is the third component, and in Eq. (1), we have chosen it as a stationary frame of reference, i.e. $J_3 \equiv 0$, and therefore not included it in the entropy production. On this basis, the corresponding flux–force relations are expressed as:

2 $$\begin{aligned} J_q^\prime =&L_{qq}\nabla \left( \frac{1}{T} \right) - \sum _{k=1}^2 L_{qk} \frac{1}{T}\nabla \mu _{k,T} \end{aligned}$$

3 $$\begin{aligned} J_j=&L_{jq}\nabla \left( \frac{1}{T} \right) - \sum _{k=1}^2 L_{jk} \frac{1}{T}\nabla \mu _{k,T}, \qquad j=1,2 \end{aligned}$$

where the phenomenological coefficients are subject to the Onsager symmetry relations, $L_{ij}=L_{ji}$. Note that even if component 3 is stagnant, it may contribute to the thermal conductivity, $L_{qq}$. However, in the present study, the matrix particles will be fixed to their lattice positions and represent a perfect thermal insulator.

We now expand $\nabla \mu _{k,T}$ into its contribution from pressure and composition:

4 $$\begin{aligned} \nabla \mu _{k,T} = V_k \nabla P + \nabla \mu _{k,c} \end{aligned}$$

where $V_k$ is the partial molar volume of component *k*. The Gibbs–Duhem equation states that

5 $$\begin{aligned} \sum _{k=1}^3 n_k (\nabla \mu _k +S_k \nabla T - V_k \nabla P) = \sum _{k=1}^3 n_k \nabla \mu _{k,c}=0 \nonumber \\ \end{aligned}$$

where $n_k$ is the mole number and $S_k$ is the partial molar entropy of component *k*. The structure of component 3 is a FCC lattice, and the concentration of matrix particles does not vary in space. Moreover, the mole fraction $x_3$ is about one order of magnitude smaller than $x_1$ and $x_2$. We therefore neglect the contribution $\nabla \mu _{3,c}$ in Eq. (5) and solve for $\nabla \mu _{2,c}$:

6 $$\begin{aligned} \nabla \mu _{2,c}= -\frac{n_1}{n_2} \nabla \mu _{1,c} \end{aligned}$$

Introduction of Eqs. (4) and (6) into Eq. (1) gives an alternative expression for the entropy production:

7 $$\begin{aligned} \sigma = J_q^\prime \nabla \left( \frac{1}{T} \right) - \frac{1}{T} J_V \nabla P - \frac{x_1}{T} J_D \nabla \mu _{1,c} \end{aligned}$$

where the mole fraction of component *i* in the fluid is $x_i=n_i/(n_1+n_2)$,

8 $$\begin{aligned} J_V = J_1 V_1 + J_2 V_2 \end{aligned}$$

is a volume flux and

9 $$\begin{aligned} J_D = \frac{J_1}{x_1} - \frac{J_2}{x_2} \end{aligned}$$

is a diffusion flux. The fluxes may now be expressed as:

10 $$\begin{aligned} J_q^\prime =&L_{qq}\nabla \left( \frac{1}{T} \right) - L_{qV} \frac{1}{T} \nabla P - L_{qD} \frac{x_1}{T} \nabla \mu _{1,c} \end{aligned}$$

11 $$\begin{aligned} J_V=&L_{Vq}\nabla \left( \frac{1}{T} \right) - L_{VV} \frac{1}{T} \nabla P - L_{VD} \frac{x_1}{T} \nabla \mu _{1,c} \end{aligned}$$

12 $$\begin{aligned} J_D=&L_{Dq}\nabla \left( \frac{1}{T} \right) - L_{DV} \frac{1}{T} \nabla P - L_{DD} \frac{x_1}{T} \nabla \mu _{1,c} \end{aligned}$$

The diagonal elements of the flux–force relations represent Fourier’s law, Darcy’s law, and Fick’s law. The relations between the *L*-coefficients in Eqs. (10)–(12) and Eqs. (2)–(3) is given in “Appendix A”.

The thermal force is expressed by $\nabla \left( 1/T \right) =- \nabla T/T^2$. For the present purpose, we set $J_1=J_2=0$ and consequently $J_V=J_D=0$, and find conditional relations between the thermodynamic forces, which lead to the following definition of the Soret coefficient:

13 $$\begin{aligned} S=&\left( \frac{\nabla x_1}{x_1x_2\nabla T} \right) _{J_V=J_D=0} \end{aligned}$$

14 $$\begin{aligned} =&\frac{1}{x_1x_2 RT^2}\left( \frac{L_{DV} L_{Vq} - L_{VV} L_{Dq}}{L_{DD}L_{VV}-L_{DV}^2} \right) \left( 1+\frac{\partial \ln \gamma _1}{\partial \ln x_1} \right) ^{-1} \end{aligned}$$

where $\gamma _1$ is the activity coefficient of component 1. The two-component isotope mixture we consider in this work is an ideal mixture in bulk fluid with $\gamma _1=1$. Equation (13) will give the sign of the Soret coefficient such that the lighter component 1 in this case migrates to the hot side of the system (the isotope effect). In the porous medium, however, the matrix will make the fluid mixture non-ideal if the two fluid components interact differently with the matrix.

Likewise, the thermo-osmotic coefficient is defined as:

15 $$\begin{aligned} D_\mathrm{P} =&\left( \frac{\nabla P}{\nabla T} \right) _{{J_V=J_D=0}} \end{aligned}$$

16 $$\begin{aligned} =&-\frac{1}{T}\left( \frac{L_{DD} L_{Vq} - L_{DV} L_{Dq}}{L_{DD}L_{VV} - L_{DV}^2} \right) \end{aligned}$$

If we use Eqs. (11) and (12) to express the forces $\nabla P$ and $\nabla \mu _{1,c}$ in terms of the fluxes $J_V$ and $J_D$ and use the results in the heat flux, Eq. (10), we get

17 $$\begin{aligned} J_q^\prime =L_{qq}\nabla \left( \frac{1}{T} \right) + q_V^* J_V + q_D^*J_D \end{aligned}$$

where the heats of transfer are

18 $$\begin{aligned} q_V^*=&\frac{L_{qV}L_{DD} - L_{qD}L_{DV}}{L_{VV}L_{DD}-L_{DV}^2} \end{aligned}$$

19 $$\begin{aligned} q_D^*=&\frac{L_{qD}L_{VV}-L_{qV}L_{DV}}{L_{VV}L_{DD}-L_{DV}^2} \end{aligned}$$

Note that $q_V^*$ and $q_D^*$ have different dimensions, see “Appendix B”. The Soret coefficient and the thermo-osmotic coefficient are related to $q_D^*$ and $q_V^*$ by

20 $$\begin{aligned} S=&-\frac{q_D^*}{x_1x_2 RT^2}\left( 1+\frac{\partial \ln \gamma _1}{\partial \ln x_1} \right) ^{-1} \end{aligned}$$

21 $$\begin{aligned} D_\mathrm{P} =&-\frac{q_V^*}{T} \end{aligned}$$

The Soret coefficient can now be interpreted as the heat of transfer conjugate to the diffusion flux and the thermo-osmotic coefficient as the heat of transfer conjugate to the volume flux.

If we express the heat flux in Eq. (2) with heats of transfer and mass fluxes, $q_1^*$ and $q_2^*$, we get

22 $$\begin{aligned} J_q^\prime = L_{qq}\nabla \left( \frac{1}{T} \right) + q_1^* J_1 + q_2^*J_2 \end{aligned}$$

with the following relations:

23 $$\begin{aligned} q_V^*=&\frac{q_1^* x_1 + q_2^* x_2}{V_1x_1+V_2x_2} \end{aligned}$$

24 $$\begin{aligned} q_D^*=&x_1x_2 \frac{q_1^* V_2 - q_2^* V_1}{V_1x_1+V_2x_2} \end{aligned}$$

or

25 $$\begin{aligned} q_1^*=&\frac{q_D^*}{x_1}+q_V^*V_1 = - x_2 RT^2\left( 1+\frac{\partial \ln \gamma _1}{\partial \ln x_1} \right) S - V_1 T D_\mathrm{P} \end{aligned}$$

26 $$\begin{aligned} q_2^*=&-\frac{q_D^*}{x_2}+q_V^*V_2 = x_1 RT^2\left( 1+\frac{\partial \ln \gamma _1}{\partial \ln x_1} \right) S - V_2 T D_\mathrm{P} \end{aligned}$$

To further illustrate the meanings of $q_V^*$ and $q_D^*$, we set the two fluid component parameters equal, so that the system becomes a quasi-one-component system with specie labels being the only difference. In this case, which will be referred to as the “color case”, $q_1^*=q_2^*$, $V_1=V_2$, and $S=0$. Equations (23), and (24) then reduce to

27 $$\begin{aligned} q_V^*&= \frac{q_1^*}{V_1} = \; \frac{q_2^*}{V_2} \end{aligned}$$

28 $$\begin{aligned} q_D^*&= x_1x_2 (q_1^* - q_2^*) = \; 0 \end{aligned}$$

which means that there is a thermo-osmotic effect, but no separation of components.

**Fig. 1:**
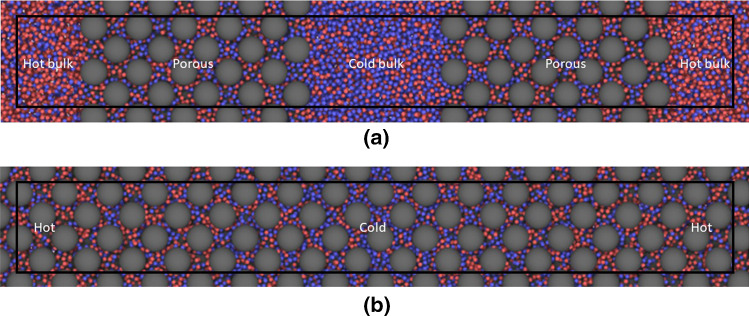
The two configurations of the porous medium used in this work. This illustration shows two cases with $\phi \approx 0.69$ and $\varepsilon _{13}^*=\varepsilon _{23}^*=1.0$, Series A (**a**) and B (**b**). The box boundaries are shown with the black line. Parts of the periodic images of the mother box are also shown. In **a** each half of the MD box is about 60% filled with the porous material and 40% with bulk fluid. In **b** the matrix fills the MD box completely. The bulk regions in **a** are thermostated to hot and cold. The fluid in the corresponding regions in **b** are thermostated likewise

The thermo-osmotic effect requires computation of the pressure, which is not well defined in a porous medium. We have therefore used a matrix configuration with bulk fluid at both sides, see Fig. 1a, and computed the pressure only in the bulk regions with the standard virial method. The Soret and thermo-osmotic coefficients in Eqs. (13) and (15) were modified to

29 $$\begin{aligned} S'=\left( \frac{1}{x_1x_2} \frac{\Delta x_1}{\Delta T} \right) _{{J_V=J_D=0}} \end{aligned}$$

and

30 $$\begin{aligned} D'_\mathrm{P}=\left( \frac{\Delta P}{\Delta T} \right) _{{J_V=J_D=0}} \end{aligned}$$

respectively, where “$\Delta$” means the property value in the hot region minus that in the cold region. The computation of pressure in the bulk fluid is straightforward. However, the interface between the bulk and the matrix may create a surface which adds resistance to the heat flow and may also give an extra contribution to the Soret effect, i.e. the coupling. A surface resistance to heat flow will show up as a discontinuity in the temperature profile. To resolve this issue, we also used a porous medium that filled the whole system as shown in Fig. 1b. A difference in the temperature profiles for two otherwise identical cases will indicate a surface resistance.

## Nonequilibrium molecular dynamics simulations

The fluid and matrix particles were modelled with a Lennard–Jones spline potential, defined by Eq. (31)

31 $$\begin{aligned} u_{ij}(r)= {\left\{ \begin{array}{ll} \infty &{} \quad \text {for }r \le \Sigma _{ij} \\ 4\varepsilon _{ij}\left[ \left( \frac{\sigma _{ij} - \Sigma _{ij}}{r - \Sigma _{ij}}\right) ^{12}-\left( \frac{\sigma _{ij} - \Sigma _{ij}}{r - \Sigma _{ij}}\right) ^{6} \right] &{} \quad \text {for } \Sigma _{ij}\le r\le r_{s,ij} \\ a_{ij}(r-r_{c,ij})^2+b_{ij}(r-r_{c,ij})^3 &{} \quad \text {for }r_{s,ij} \le r\le r_{c,ij} \\ 0 &{} \quad \text {for }r\ge r_{c,ij} \end{array}\right. } \end{aligned}$$

**Fig. 2:**
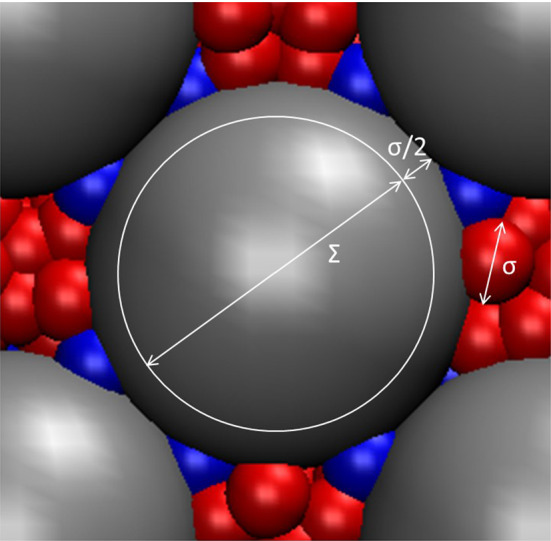
Matrix particle (grey) showing the hard-core diameter $\Sigma$ and the Lennard–Jones skin of thickness $\sigma /2$. The two fluid components (red and blue) are of equal size with diameter $\sigma$

The subscripts *ij* represent the combination of the three components, in this case the six combinations of two fluid components and one matrix component. The parameter $\Sigma _{ij}=(\Sigma _i+\Sigma _j)/2$ was introduced to give the matrix particles a hard core, in the present case $\Sigma _i=0$ for the fluid particles ($i=1,2$) and $\Sigma _3=\Sigma >0$ where $\Sigma$ is the core diameter of the matrix particles. This is illustrated in Fig. 2. The “skin thickness” parameters $(\sigma _{ij} - \Sigma _{ij})$ were all fixed to the same value, $\sigma$, in the study reported here. We have used the Lorentz–Berthelot mixing rules for the particle diameters, $\sigma _{ij}=(\sigma _i+\sigma _j)/2$. The absorption of the fluid in the matrix (wettability) was controlled with the parameters $\varepsilon _{13}$ and $\varepsilon _{23}$. A “neutral” case was made by setting all $\varepsilon _{ij}=1.0$.

The parameters $a_{ij}$, $b_{ij}$, $r_{s,ij}$, and $r_{c,ij}$ in the Lennard–Jones/spline potential are determined so as to truncate the potential smoothly from the inflection point of the full Lennard–Jones potential to zero. The parameters are given by the algebraic equations

32 $$\begin{aligned} r_{s,ij}=&\left( \frac{26}{7} \right) ^{1/6} (\sigma _{ij}-\Sigma _{ij} ) + \Sigma _{ij} = \left( \frac{26}{7} \right) ^{1/6} \sigma + \Sigma _{ij} \end{aligned}$$

33 $$\begin{aligned} r_{c,ij}=&\frac{67}{48} r_{s,ij} - \frac{19}{48} R_{ij} \end{aligned}$$

34 $$\begin{aligned} a_{ij}=&-\frac{24192}{3211} \frac{\varepsilon _{ij}}{(r_{s,ij}-\Sigma _{ij})^2} \end{aligned}$$

35 $$\begin{aligned} b_{ij}=&-\frac{387072}{61009} \frac{\varepsilon _{ij}}{(r_{s,ij}-\Sigma _{ij})^3} \end{aligned}$$

The mass ratio of the fluid particles was 10 to 1. The other parameters were $\sigma _{11}=\sigma _{22}=\sigma _{12}=\sigma$ and $\varepsilon _{11}=\varepsilon _{22}=\varepsilon _{12}=\varepsilon$; in other words, this is an isotope mixture. The overall fluid mole fraction was 0.5.

We used a tetragonal MD box with $L_y=L_z=L_x/8$. Two configurations were used as shown in Fig. 1 with the total number of particles $N=N_1+N_2+N_3=16,384$. We confirmed that this number was large enough to avoid size effects in the results. The number of matrix particles was $N_3=160$ in Series A shown in Fig. 1 and $N_3=256$ in Series B. The two-component fluid mixture was equimolar with $N_1=N_2=8114$ in Series A and $N_1=N_2=8064$ in Series B. We denote the mole fraction of matrix particles by $x_3=N_3/N$ and the volume fraction of the porous medium by $y=V_\mathrm{m}/V$ where *V* is the total volume of the MD box and $V_\mathrm{m}$ is the volume of the porous medium. In this work, $y=0.625$ and 1.0 for Series A and B, respectively. The porosity of the porous medium is the ratio between the pore volume $V_\mathrm{p}$ and $V_\mathrm{m}$:

36 $$\begin{aligned} \phi = \frac{V_\mathrm{p}}{V_\mathrm{m}}= 1- \frac{N_3 b}{V_\mathrm{m}} = 1- \frac{x}{y} \rho b \end{aligned}$$

where $\rho = N/V$ is the overall particle density in the system (including the matrix particles) and *b* is the volume of a single matrix particle. The particle volume is not well defined for a soft particle like a Lennard–Jones particle, but we have used $b=\pi \sigma _{33}^3 /6$.

The number of fluid particles in the matrix is

37 $$\begin{aligned} N_\mathrm{f}^\mathrm{m} = \rho _\mathrm{f}^\mathrm{m} V_\mathrm{p} = \rho _\mathrm{f}^\mathrm{m} \phi V_\mathrm{m} = y \rho _\mathrm{f}^\mathrm{m} \phi V \end{aligned}$$

where $\rho _\mathrm{f}^\mathrm{m}$ is the fluid density in the porous medium. The system’s porosity was controlled by the size of the matrix particles.

The number of fluid particles in the bulk is

38 $$\begin{aligned} N_\mathrm{f}^\mathrm{b} = (1-y) \rho _\mathrm{f}^\mathrm{b} V \end{aligned}$$

where $\rho _\mathrm{f}^\mathrm{b}$ is the fluid density in the bulk.

The fluid densities were determined as follows. Equilibrium simulations were performed for Series A at neutral wettability, reduced temperature $T^*=kT/\varepsilon _{11}=3.0$, and reduced fluid density in the bulk $(\rho _\mathrm{f}^{b})^* = \rho _\mathrm{f}^\mathrm{b} \sigma _{11}^3 = 0.7$. This is a typical liquid density. The corresponding fluid density in the porous medium was monitored, and the total number of fluid particles was determined from Eqs. (37) and (38) and confirmed against the total number of particles in the system. This procedure was repeated for 8 different porosities with the results shown in Table 1.

**Table 1:** Porosities and equilibrium densities at $T^*=3.0$. The $\rho _\mathrm{f}^\mathrm{b}$ refers to the bulk fluid density in Series A and $\rho _\mathrm{f}^\mathrm{m}$ refers to fluid density in the pores in both series. The density $\rho$ is the average fluid density in the entire system

Case	$\sigma _{33}/\sigma _{11}$	$\phi$	$(\rho _\mathrm{f}^\mathrm{b})^*$	$(\rho _\mathrm{f}^\mathrm{m})^*$	$\rho ^*$ (Series A)	$\rho ^*$ (Series B)
1	6.2	0.33	0.70	0.39	0.35	0.13
2	5.8	0.40	0.70	0.42	0.37	0.17
3	5.4	0.47	0.70	0.50	0.41	0.24
4	5.0	0.54	0.70	0.54	0.45	0.30
5	4.2	0.69	0.70	0.59	0.52	0.41
6	3.8	0.75	0.70	0.62	0.56	0.47
7	3.0	0.86	0.70	0.65	0.62	0.57
8	2.0	0.96	0.70	0.68	0.67	0.66

Series B was used with the same porosities and fluid densities in the porous medium as in Series A. The different overall densities is due to the contribution from the bulk in Series A, which is not relevant in Series B.

The system was divided in *x*-direction into 64 layers of equal thickness. A temperature gradient was created in Series A by thermostating the fluid particles in the bulk (five layers) at each end of the MD box to a uniform high temperature $T_\text {H}^*=4.0$ and in the middle (ten layers) to a uniform low temperature $T_\text {L}^*=2.0$ by velocity scaling. Also in Series B, five layers at each end and ten layers in the center of the MD box were used for thermostating the fluid, but the thermostat was applied to the fluid particles only. The average fluid temperature in the entire system was approximately equal to 3.0 (in reduced units), which is slightly higher than three times the critical temperature for this fluid.

We were also interested in the effect of wettability preferences; does a difference in wettability between fluid and matrix have an effect on the Soret coefficient? This was studied by varying the energy parameters between the fluid and the matrix, $\varepsilon _{13}$ and $\varepsilon _{23}$, such that the interaction between the lighter component (component 1) and the matrix was stronger in some cases and opposite in other cases. This is quantified by the difference $\varepsilon _{23}-\varepsilon _{13}$. The larger this difference is, the more do the heavy particles wet the matrix particles. For each case, 11 different values of $\varepsilon _{23}^*-\varepsilon _{13}^*$ with intervals 0.2 were used in the range between − 1.0 and $+$ 1.0 ($\varepsilon _{i3}^* \equiv \varepsilon _{i3}/\varepsilon _{11}$, $i=1,2$).

A summary of the parameter values is given in Table 2.

**Table 2:** Parameter values used in the MD simulations

Parameter	Value	Meaning
$(\rho _\mathrm{f}^\mathrm{b})^*$	0.7	Fluid density in the bulk (case *a*)
$T_\mathrm{H}^*$	4.0	High thermostat set point
$T_\mathrm{L}^*$	2.0	Low thermostat set point
$m_2/m_1$	10.0	Fluid particle mass ratio
$\varepsilon _{22}/\varepsilon _{11}$	1.0	Potential depth ratio
$\sigma _{22}/\sigma _{11}$	1.0	Fluid particle diameter ratio
$x_1$	0.5	Mole fraction of fluid component 1
$\phi$	0.33 to 1.0	Matrix porosity
$\varepsilon _{23}^*-\varepsilon _{13}^*$	− 1.0 to $+$ 1.0	Wettability preference. Higher value means
		heavy particles are more wetting.
$\sigma _{33}/\sigma _{11}$	2.0 to 6.2	Matrix particle size

For each case, five parallel runs were made, each starting from a FCC structure. The matrix particles were frozen to their lattice positions. Prior to the MD runs, the fluid particle positions were randomized with different number of Monte Carlo steps. The number of MD steps was $3 \times 10^7$ for each parallel run with the last $5 \times 10^6$ steps used for data acquisition. The mass fluxes were monitored, which showed that the large number of steps was necessary to reach steady state, especially for lowest porosities.

A snapshot of the system in Case 5, Series A and B, is shown in Fig. 1. The illustration shows a surplus of heavy (blue) particles in the middle of the MD box where the temperature is low and red (light) particles at the ends where the temperature is high.

Data for temperature and composition were recorded and analyzed according to Eqs. (13) and (29) for the Soret coefficient and with Eq. (30) for the thermo-osmotic coefficient. The difference, such as $\Delta x_1$, is the value in the hot region minus that in the cold region. The configuration used in Series A introduces an interface between bulk and matrix. The fluid density in the matrix is lower than in the bulk and this change in density may give an additional resistance to the heat flux (the Kapitza resistance). The matrix particles do not conduct heat in our case. The change in fluid density has no effect on the mass fluxes, which are zero at the steady state we shall consider.

## Results

### The Soret coefficient

#### Porosity effects

**Fig. 3:**
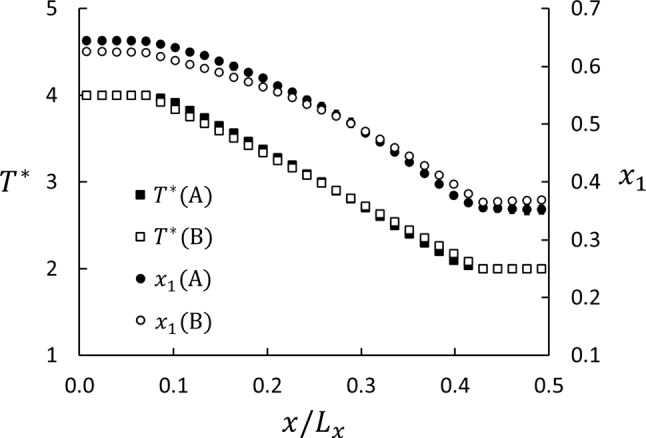
Profiles of mole fraction and temperature for Series A and B, Case 5 (cf. Table 3). The abscissa is in units of MD box length in *x*-direction. The errors, determined as three standard errors based on data from five parallel runs, are about the size of the symbols

A typical example of mole-fraction and temperature profiles at steady state with $J_1=J_2=0$ is shown in Fig. 3. These results are for the cases shown in Fig. 1. The five points at each end of the graphs represent the thermostated layers in the MD box. These are the regions with the bulk fluid in Series A. The temperature profiles are linear in the matrix, and the mole-fraction profile deviates slightly from linearity. The slope of the temperature profile in Case B5 shifts abruptly from zero to negative between the thermostated and not-thermostated layers, whereas the profile in Case A5 shows a slightly smoother transition between the thermostated and non-thermostated regions. However, there appears to be no Kapitza resistance in Series A, which would have shown up as a discontinuity in the temperature profiles across the surfaces.

Such data were used to compute $S'$ from Eq. (29) with the results listed in Table 3. The data for Series A and B show the same trend as function of porosity, but they differ by more than the combined statistical errors for $\phi < 0.7$. We also determined *S* in the central part of the matrix using Eq. (13). The ratio $\nabla x_1/\nabla T$ was determined from a plot of $x_1$ versus *T*, and the ratio was determined as the slope. Only the 14 middle layers in the matrix were used in this analysis to avoid the direct impact of the surfaces.

**Fig. 4:**
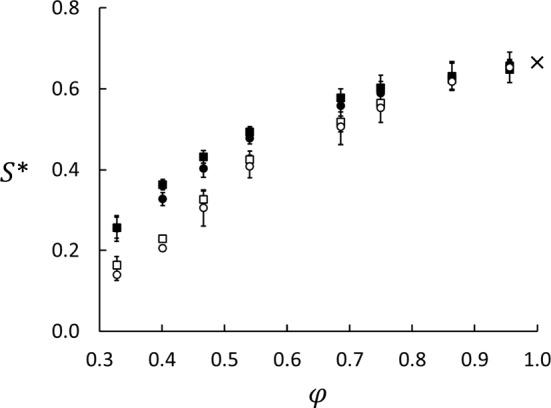
Soret coefficients for the neutrally wetting case ($\varepsilon _{23}^*-\varepsilon _{13}^*=0$) as function of porosity. Series A (black) and B (white) are for the systems shown in Fig. 1a and b, respectively. The Soret coefficients were computed in two ways for each series, the squares from Eq. (29) using the difference between the thermostated regions. The circles from Eq. (13) using the gradients in the central part of the matrix. The uncertainties are three standard errors. The cross at $\phi =1.0$ shows the result for bulk a fluid ($S^* =0.666 \pm 0.005$)

**Table 3:** Values for $\Delta x_1$ and $S'^*$ from Series A and B computed with Eq. (29). In all cases, $\Delta T^* = 2.0$ and $\varepsilon _{23}^*-\varepsilon _{13}^*=0$

Case	$\phi$	$\Delta x_1$ (Series A)	$S'^*$ (Series A)	$\Delta x_1$ (Series B)	$S'^*$ (Series B)
1	0.33	$0.13 \pm 0.01$	$0.26 \pm 0.03$	$0.082 \pm 0.01$	$0.16 \pm 0.02$
2	0.40	$0.182 \pm 0.01$	$0.36 \pm 0.01$	$0.114 \pm 0.003$	$0.23 \pm 0.01$
3	0.47	$0.216 \pm 0.008$	$0.43 \pm 0.02$	$0.163 \pm 0.01$	$0.33 \pm 0.02$
4	0.54	$0.246 \pm 0.007$	$0.49 \pm 0.01$	$0.213 \pm 0.01$	$0.43 \pm 0.02$
5	0.69	$0.29 \pm 0.01$	$0.58 \pm 0.02$	$0.259 \pm 0.01$	$0.52 \pm 0.02$
6	0.75	$0.30 \pm 0.01$	$0.60 \pm 0.03$	$0.283 \pm 0.008$	$0.57 \pm 0.02$
7	0.86	$0.32 \pm 0.02$	$0.63 \pm 0.04$	$0.311 \pm 0.004$	$0.62 \pm 0.01$
8	0.96	$0.324 \pm 0.003$	$0.65 \pm 0.01$	$0.328 \pm 0.006$	$0.66 \pm 0.01$

The results determined from Eqs. (13) and (29) for Series A and B for $\varepsilon _{13}^*=\varepsilon _{23}^*=1.0$ are shown in Fig. 4. Whether we used the difference between the properties in the thermostated regions or the gradients had little effect on the values of the Soret coefficient in this example, the results agreed within the combined statistical errors. However, we did find a significant difference between Series A and B as shown in Table 3 and Fig. 4. This difference can only be explained by the difference in the boundary conditions for the two series, as will be further discussed in Sect. 5.

**Fig. 5:**
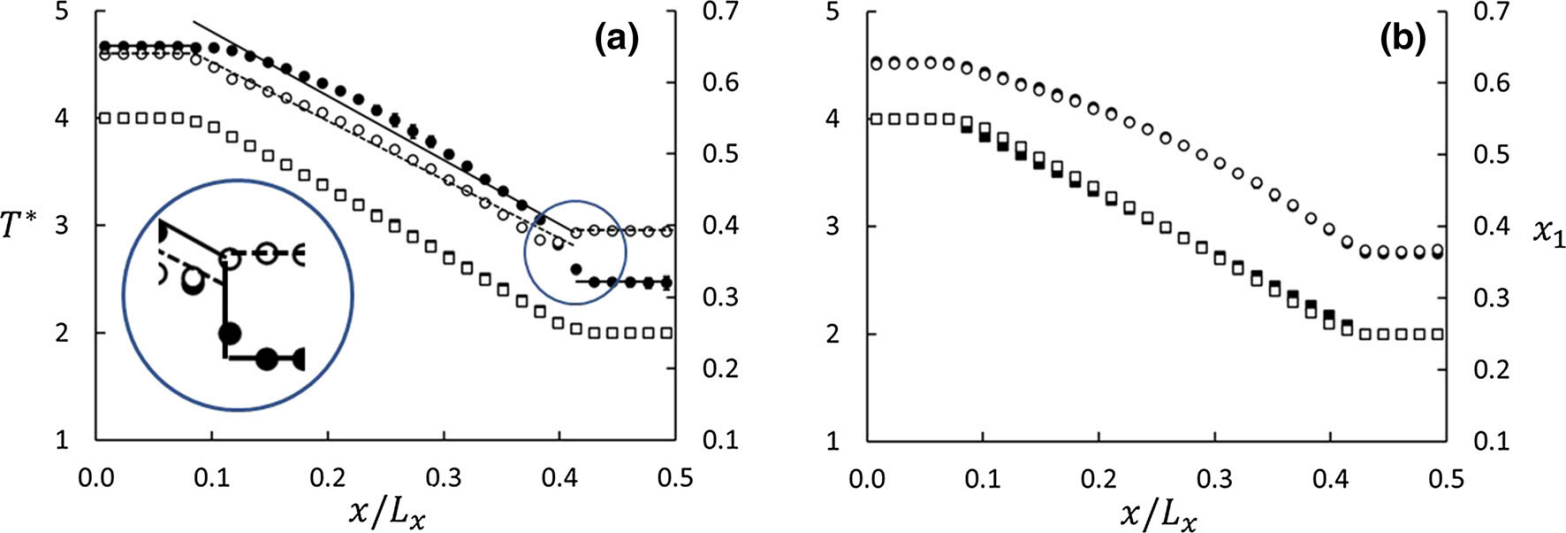
Profiles of mole fraction (circles, right axis) and temperature (squares, left axis) for Series A (graph **a**) and B (graph **b**) at $\phi =0.69$. The black and white symbols are for $\varepsilon _{23}^*-\varepsilon _{13}^*=-1.0$ (lighter component more wetting) and $+1.0$, respectively. If the plots overlap, only white symbols are shown. The abscissa is in units of MD box length in *x*-direction. The errors in both $x_1$ and $T^*$, based on data from five parallel runs, are represented by the symbol size. The insert in panel (**a**) shows details of the jump in mole-fraction profiles between the matrix and bulk on the cold side. The guidelines are linear fits to the data in the matrix and the bulk

**Fig. 6:**
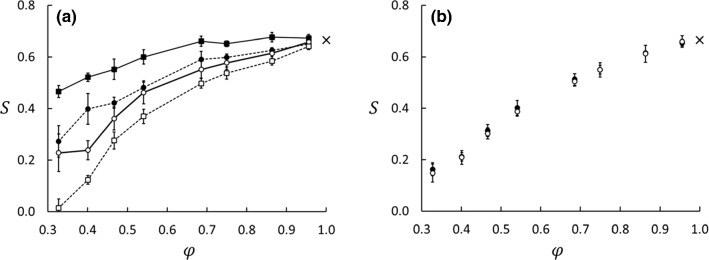
Effect of wettability preference on the Soret coefficient for the equimolar mixture in Series A (**a**) and B (**b**). The Soret coefficients in Series A were computed from Eqs. (13) and (29) and are shown as circles and squares, respectively. The Soret coefficients in Series B were computed from Eq. (13) only. Black and white symbols are for $\varepsilon _{23}^*-\varepsilon _{13}^*=-1$ and $+1$, respectively. The cross at $\phi =1.0$ is the result for the bulk fluid. The errors bars are based on data from five parallel runs with randomized initial configurations

#### Wettability effects

The wettability preference was controlled by the parameters $\varepsilon _{13}^*$ and $\varepsilon _{23}^*$ such that the difference $\varepsilon _{23}^*-\varepsilon _{13}^*$ was varied in 11 steps between − 1 and $+$ 1. Temperature- and mole fraction profiles for two extreme cases, $\varepsilon _{23}^*-\varepsilon _{13}^*= -1$ and $+1$ for Case 5, $\phi =0.69$, are shown in Fig. 5. In Series A, with the bulk fluid reservoirs, the absorption of the wetting component in the matrix led to a deficit of the same component in the bulk compared with the neutrally wetting case (Fig. 3). This deficit was non-symmetric in the sense that it was larger in the cold bulk phase than in the hot, indicating a stronger absorption of the wetting component at the lower temperature. The result was a large distortion of the Soret coefficient as computed from Eq. (29). This way of computing the Soret coefficient therefore gave a dramatic effect of the wettability preference as shown in Fig. 6a. When the lighter component 1 was more wetting ($\varepsilon _{23}^*-\varepsilon _{13}^*= -1$), the deficit in the cold region worked in the same direction as the isotope effect, leading to a larger apparent Soret coefficient than in the neutral case. On the contrary, when the heavier component 2 was more wetting ($\varepsilon _{23}^*-\varepsilon _{13}^*= +1$), the absorption effect worked in the opposite direction, leading to a smaller apparent Soret coefficient than in the neutral case. In the matrix, the mole-fraction profiles showed approximately the same gradients, irrespective of the wettability preference. When computed from Eq. (13), using the temperature- and mole fraction data for the central part of the matrix, the Soret coefficient appeared to be less sensitive to the wettability preference (see Fig. 6a). This was confirmed by data from Series B, where we again used mole-fractions and temperatures from the central part of the matrix and Eq. (13). The profiles were found to be independent of the wettability preference, and so was the Soret coefficient, see Fig. 6b.

**Fig. 7:**
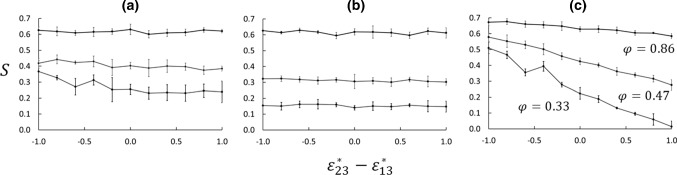
Soret coefficient as function of wettability preference. All panels show data for $\phi =0.33, 0.47, 0.86$. **a** is for Series A with Eq. (13), **b** for Series B with Eq. (13), and **c** for Series A with Eq. (29). The errors bars are based on data from five parallel runs

Results for the Soret coefficient as function of wettability preference are given in Fig. 7. When *S* is computed from the gradients of *T* and $x_1$ in the porous medium, using Eq. (13), the wettability preference has little effect on the Soret coefficient, except for the lowest porosities in Series A. The values are slightly smaller for Series B than for A for the lower porosities, which is also clear from Fig. 4. When $S'$ is computed from the difference between the bulk fluid values in Series A, however, there is a dramatic effect of the wettability preference, especially for the lower porosities due to the absorption effect discussed above. These results have some serious implications on experimental designs made to measure properties of porous media. We have here demonstrated that the system layout is decisive for the outcome of the result, and can lead to deviations as pictured in Fig. 4.

### Thermo-osmosis

**Fig. 8:**
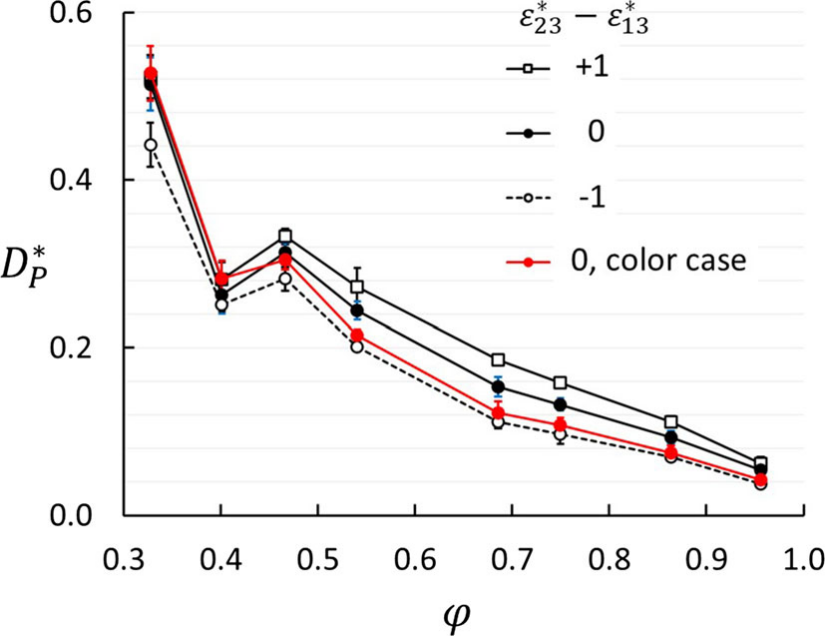
Thermo-osmotic coefficient as function of porosity for three values of the wettability preference $\varepsilon _{23}^* - \varepsilon _{13}^*$. The red symbols show results for $\varepsilon _{23}^* = \varepsilon _{13}^*$ and $m_2=m_1$, i.e. the “color” case with no Soret effect

The thermo-osmotic coefficient was determined from Series A using Eq. (30). We found a pressure build-up at the hot side of the porous medium, $D_\mathrm{P}>0$. Selected results are shown in Fig. 8. The thermo-osmotic coefficient decreases significantly with increasing porosity and tends to zero as the porosity tends to one, but with an irregularity around $\phi =0.4$. We will show later on that this irregularity correlates with an irregularity in the absorption in the matrix. There is a clear, but smaller dependency on the wettability preference. The results from the color case agree well with the other results. Since the composition is irrelevant for $D_\mathrm{P}$ in the color case, this means that $D_\mathrm{P}$ depends weakly on the composition.

**Fig. 9:**
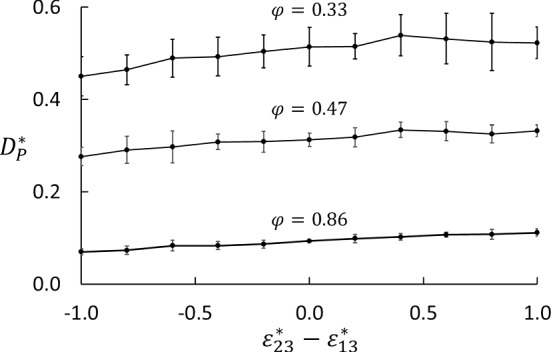
Thermo-osmotic coefficient as function of wettability preference $\varepsilon _{23}^* - \varepsilon _{13}^*$ for three porosities

The effect of the wettability preference is shown in Fig. 9. We found a slight, but significant increase in $D_\mathrm{P}$ with increasing wettability of the heavy component. The trends were the same for all porosities, but most clear for the highest porosity.

### Fluid absorption and composition in the porous medium

**Fig. 10:**
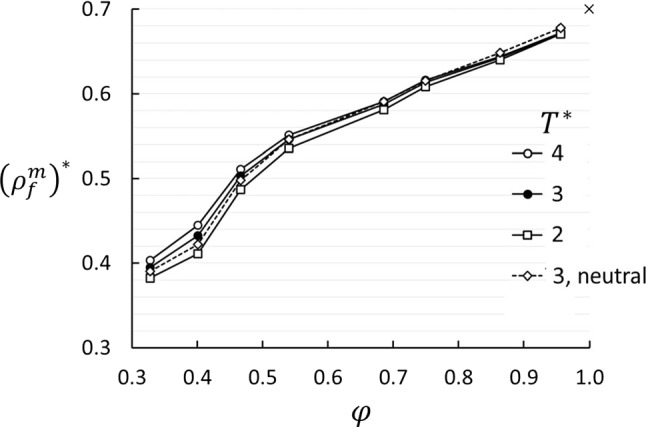
Fluid density in the matrix as function of porosity for three temperatures. The case “3, neutral” is for $\varepsilon _{23}^*-\varepsilon _{13}^*=0$, the others are for $\varepsilon _{23}^*-\varepsilon _{13}^*=-1$. The cross at $\phi =1.0, \left( \rho _\mathrm{m}^f \right) ^*=0.7$ marks the bulk value. Errors are shown as symbol size

Equilibrium simulations were made to clarify the effect of wettability preference in the porous medium. Figure 10 shows the fluid density in the pore volume as function of porosity. The density increases with increasing porosity, which means that the fluid particles can pack more densely for larger pore volumes. The effect of temperature is small, and there is no apparent effect of the wettability preference. Note the wavy behavior for $\phi < 0.5$, which coincides with the dip in $D_\mathrm{P}$ at the same porosity.

**Fig. 11:**
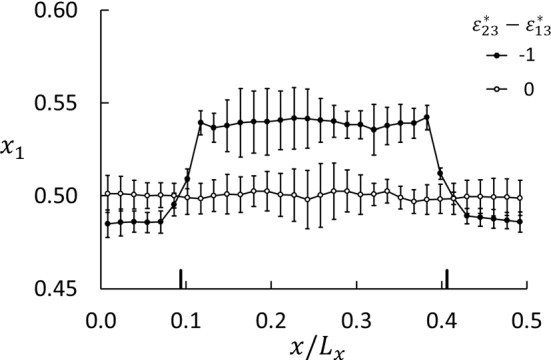
Profiles of the mole fraction of component 1 in equilibrium simulations at $T^*=3$ and $\phi =0.33$ for two wettability preferences. The two thick vertical lines on the *x*-axis mark the limits of the porous medium ($0.09< x/L_x < 0.41$). These mole fractions represent the fluid composition only, not including the matrix particles. The error bars are based on five runs with different initial configurations

Another aspect of the fluid distribution in the matrix is the composition. Figure 11 shows two profiles of $x_1$ for $\varepsilon _{23}^*-\varepsilon _{13}^*=-1$ and 0 at $T^*=3$.

**Fig. 12:**
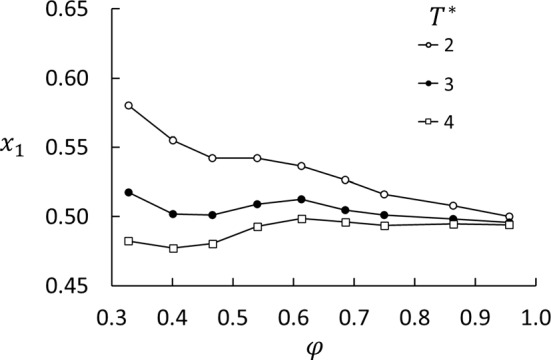
Corrected mole fraction of component 1 in the porous medium as function of porosity for $\varepsilon _{23}^*-\varepsilon _{13}^*= -1$ (component 1 more wetting) for three temperatures. The mole fraction has been corrected for the presence of matrix particles, which means that $x_1+x_2 < 1.0$

The mole fraction shown in the figure is based on the two-component fluid with $x_1+x_2=1$. In the porous medium, this is not quite correct because the matrix (component 3) must also be included. With 160 matrix particles, the correction is small, $\Delta x_1 \approx -160 x_1/N_\text {f}^\mathrm{m}$ where $N_\text {f}^\mathrm{m}$ is the number of fluid particles in the matrix, which varies from 4000 to 10,000 for porosities varying from 30 to 100%. The mole fractions shown in Fig. 12 were corrected in this way. In the neutral case ($\varepsilon _{23}^*-\varepsilon _{13}^*=0$), the fluid composition is approximately the same, $x_1=0.5$, in the porous medium as in the bulk. When the lighter component 1 is more wetting ($\varepsilon _{23}^*-\varepsilon _{13}^*=-1$), this component is enriched in the porous medium and depleted in the bulk. When the heavier component 2 is more wetting, the effect is opposite. The difference in mass of the two components has no effect on the component distribution in these equilibrium cases and is a consequence of the porous medium only.

All equilibrium data for the mole fraction are shown in Fig. 12. At $T^*=4$, which is the temperature at the hot side used in the non-equilibrium simulations of *S* and $D_\mathrm{P}$, component 1 is almost uniformly distributed. On the cold side, at $T^*=2$, component 1 is much more abundant. This explains the step in $x_1$ shown in Fig. 5 at the cold side, while the hot side does not show a step. The consequence for the Soret coefficient is shown in Fig. 6a. For all temperatures, we found an oscillatory behavior of $x_1$ superposed on a decaying backbone, with a minimum around $\phi \approx 0.4$ and a weaker maximum around $\phi \approx 0.6$, and possibly a weak minimum around $\phi \approx 0.8$.

**Table 4:** Activity coefficients for components 1 and 2 in the porous medium for $\varepsilon _{23}^*-\varepsilon _{13}^*=-1$. The uncertainties are $\pm 2$ in the last digit

Porosity	$\gamma _1$			$\gamma _2$		
	$T^*=2$	$T^*=3$	$T^*=4$	$T^*=2$	$T^*=3$	$T^*=4$
0.33	0.803	0.940	1.033	1.409	1.161	1.051
0.40	0.844	0.978	1.051	1.292	1.096	1.018
0.47	0.862	0.978	1.046	1.236	1.081	1.010
0.54	0.851	0.951	1.007	1.240	1.103	1.041
0.61	0.868	0.948	0.991	1.208	1.103	1.054
0.69	0.882	0.962	0.995	1.180	1.082	1.046
0.75	0.914	0.974	1.005	1.135	1.066	1.032
0.86	0.941	0.984	1.002	1.099	1.052	1.032
0.96	0.975	0.997	1.004	1.059	1.036	1.028

The activity coefficient of the two fluid components in the porous medium was determined from system A as follows. At equilibrium, the activity of each fluid component, $a_i=x_i \gamma _i$, is the same in the bulk as in the matrix, hence

39 $$\begin{aligned} \gamma _i^\text {m}=\frac{x_i^\text {b}}{x_i^\text {m}} \gamma _i^\text {b} \end{aligned}$$

where superscripts “b” and “m” mean “in bulk” and “in matrix”, respectively. The activity coefficients in the bulk phases are unity because the isotope mixture is ideal. Results for $\gamma _1$ and $\gamma _2$ for $\varepsilon _{23}^*-\varepsilon _{13}^*=-1$ (component 1 more wetting) are given in Table 4. Due to the symmetry of the system, the values for $\gamma _1$ are valid for $\gamma _2$ when $\varepsilon _{23}^*-\varepsilon _{13}^*=+1$, and the values for $\gamma _2$ in the table are valid for $\gamma _1$ when $\varepsilon _{23}^*-\varepsilon _{13}^*=+1$. The activity coefficients are all close to unity, except at the lower porosities and temperature.

## Discussion

Colombani et al. [22] found that the Soret coefficient decreased with decreasing porosity down to $\phi =0.75$ (the lowest porosity they considered). Our results show that this trend continues at lower porosities. At $\phi =0.33$, the Soret coefficient computed in Series A is about one third of the bulk fluid value and from Series B about one quarter. It is known that the Soret coefficient depends on other molecular parameters, which will certainly be the case also in porous media, but it is clear that the porous medium itself has an effect on the coefficient. Our results do therefore not support early findings that the porous medium does not affect the measured values [19–21].

We found that the two configurations of the Soret cell shown in Fig. 1 give very different values of the Soret coefficient. With bulk fluid on the sides of the porous medium (System A), we got larger values than in System B without such a bulk fluid (Fig. 4). This shows that if a packed column has bulk fluid outside the packing, it is important to specify how the sampling is done. Both systems are closed, which means that Series A, unlike Series B, has fluid reservoirs that are open only to the porous medium. The fluid density, fluid–matrix interactions, and to some extent the composition change across the bulk/matrix boundaries. A mass flux will change not only the composition in the porous medium, but also in the bulk. Therefore, the capacity of the reservoir will influence the measurements. We also found that the preferential absorption of the two components had a dramatic effect on the Soret coefficient in Series A, but not in Series B. This has two important implications. (1) When the Soret coefficient is measured with the packed column method and fluid samples are drawn from outside the porous medium, the values may be distorted by the wettability. (2) The Soret effect may depend on variations in the wettability.

The values for the Soret coefficient shown in Figs. 4 and 6 can be converted to SI units by using a methane/decane mixture as an example. The conversion from reduced Lennard–Jones units to SI units is given in “Appendix B”. Although we consider only the mass difference and neglect the large difference in molecular size, the example may still serve as a rough guide. We choose the lighter component, methane, as component 1, with $\sigma _{11} = 3.5 \times 10^{-10}$ m and $\varepsilon _{11}/k_\text {B}=150$ K. A value in bulk fluid, $S^*=0.67$ gives $S=4.5 \times 10^{-3} \text { K}^{-1}$, which is the same order of magnitude as found in previous studies [32]. At 30% porosity, this value is reduced to about $1.3 \times 10^{-3} \text { K}^{-1}$. A mixture with $x_1=0.5$ will at 30% porosity show a separation to $x_1 \approx 0.502$ at the hot side and $x_1 \approx 0.498$ at the cold side for a temperature difference $\Delta T = 10$ K.

The main reason why System A was used, was the wish to investigate the thermo-osmotic coefficient. The definition and computation of pressure in a porous medium are topics of discussion [33, 34], so we have used the classic virial route to get the pressure in the bulk fluid only. Moreover, the thermo-osmotic pressure is, like the normal osmotic pressure, measured as a pressure difference over a membrane, not in the membrane itself.

It is known that the thermo-osmotic coefficient can be both positive and negative [23]. We found a positive coefficient, i.e. the thermo-osmotic flux direction is from cold to hot. The $D_\mathrm{P}$ is directly related to the heat of transfer $q_V^*$ by Eq. (21), so it must be related to differences in the enthalpy across the porous medium. The difference in molar enthalpy at the hot side minus that at the cold side is positive in the system we have studied, but we also have to consider the change in enthalpy across the interfaces between bulk and porous medium. Additional studies are necessary to elaborate this point. In bulk fluid, the pressure is uniform ($D_\mathrm{P}=0$), but in a porous medium or a membrane, the pressure difference can be quite high. The highest value we found, $D_\mathrm{P}^* \approx 0.5$ at 30% porosity, corresponds to $1.5 \times 10^5$ Pa/K in SI units for a two-component mixture like methane/decane. This is the same order of magnitude as has been measured in laboratory experiments of frost heave [35].

We found that the thermo-osmotic coefficient depends weakly on the difference in interaction strength between fluid and matrix particles ($\varepsilon _{23}^*-\varepsilon _{13}^*$), see Figs. 8 and 9. Kedem and Katchalsky [36] argued that if a membrane is non-selective (i.e. $\varepsilon _{23}^* - \varepsilon _{13}^* =0$), then $L_{DV}=0$. The weak dependency of $D_\mathrm{P}$ on ($\varepsilon _{23}^* - \varepsilon _{13}^*$) shown in Fig. 9 suggests that $L_{DV}$ is small also for $\varepsilon _{23}^* - \varepsilon _{13}^* \ne 0$. If we expand $D_\mathrm{P}$ in Eq. (16) in powers of $L_{DV}$, we get to first order

40 $$\begin{aligned} D_\mathrm{P} \approx -\frac{1}{T}\left( \frac{L_{Vq}}{L_{VV}} - \frac{L_{Dq}}{L_{DD}}\frac{L_{DV}}{L_{VV}}\right) \end{aligned}$$

Similarly, expanding Eq. (14), the same way gives

41 $$\begin{aligned} S \approx -\frac{1}{x_1x_2 RT^2}\left( \frac{L_{Dq}}{L_{DD}} - \frac{L_{Vq}}{L_{VV}} \frac{L_{DV}}{L_{DD}} \right) \left( 1+\frac{\partial \ln \gamma _1}{\partial \ln x_1} \right) ^{-1} \nonumber \\ \end{aligned}$$

Considering $D_\mathrm{P}$ as a linear function of $L_{DV}/L_{VV}$ and *S* as a linear function of $L_{DV}/L_{DD}$ to this first order, we find that the constant term in $D_\mathrm{P}$ is the coefficient of the linear term in *S* (apart from factors of mole fractions and temperature) and vice versa. This shows how *S* and $D_\mathrm{P}$ are coupled. Since *S* and $D_\mathrm{P}$ are both positive in this case, $L_{Vq}/L_{VV}$ and $L_{Dq}/L_{DD}$ must both be negative. Figure 9 shows a positive trend for $D_\mathrm{P}$ as function of $\varepsilon _{23}^* - \varepsilon _{13}^*$, which means that an increase in $\varepsilon _{23}^* - \varepsilon _{13}^*$ gives a decrease in $L_{DV}/L_{DD}$. Similarly, Fig. 7a shows a slight negative trend for *S* as function of $\varepsilon _{23}^* - \varepsilon _{13}^*$, which means that an increase in $\varepsilon _{23}^* - \varepsilon _{13}^*$ gives an increase in $L_{DV}/L_{VV}$.

Finally, we shall point at a relation between the Soret effect in porous media and bulk fluids. Consider the zeroth-order term in Eq. (41). For a bulk two-component fluid mixture, the Soret coefficient is defined as the ratio between the thermodiffusion and molecular diffusion coefficients (see, e.g., Platten [37]),

42 $$\begin{aligned} S_\text {bulk}=\frac{D_T}{D} \end{aligned}$$

where $D_T$ and *D* are related to the coefficients $L_{1q}$ and $L_{11}$ for binary fluid mixtures [38]. By analogy, we may define

43 $$\begin{aligned} D_T=-\frac{L_{Dq}}{x_1 x_2 T^2} \end{aligned}$$

and

44 $$\begin{aligned} D=\frac{x_1}{T} L_{DD} \frac{\partial \mu _1}{\partial x_1} \end{aligned}$$

which gives

45 $$\begin{aligned} S_{(L_{DV}=0)} = \frac{D_T}{D} \end{aligned}$$

for the porous system. The condition $(L_{DV}=0)$ means that Eq. (45) applies to a non-selective membrane. For a non-selective membrane, $L_{Dq}$ is analogous to $L_{1q}$ for binary mixtures and $L_{DD}$ is analogous $L_{11}$.

The dip in $D_\mathrm{P}$ at $\phi \approx 0.4$ is somewhat mysterious. It is almost independent of the wettability preferences (including neutral). The color case shows the same dip. This means that the observed dip in $D_\mathrm{P}$ is not a consequence of non-ideal mixture behavior. Nor is it a function of the interaction between the fluid and the matrix. The fluid density in the matrix shows a slightly irregular trend around the same porosity (Fig. 10). It must therefore be caused by factors that are unrelated to the wettability preference. We have speculated that it is a consequence of packing of fluid particles in tetrahedral and octahedral voids in the lattice of matrix particles. Since the matrix particles are at fixed positions, the size of the voids will increase in a regular manner with increasing porosity. For instance, the diameter of an inscribed sphere in an octahedral hole at $\phi =0.4$ is approximately three times the diameter of the fluid particle. Another possible explanation is related to the balance between the thermal and hydrostatic forces at steady state. The temperature gradient will drive the fluid from cold to hot along the matrix particle surfaces due to the gradient in surface tension [39], and the pressure build-up will drive the fluid back in the open pores. This balance may be shifted one way or the other, depending on the porosity and the pore structure, and so give an irregular behavior of $D_\mathrm{P}$. A more detailed analysis of the packing of fluid particles in the matrix will be necessary to resolve this question.

## Conclusions

In this work, we have used non-equilibrium thermodynamics to show that a temperature difference across a binary mixture of isotopes in a porous medium does not only lead to separation of components, but also to a pressure difference over the medium. The first observation is well known from studies of Soret equilibria. The other effect, called thermo-osmosis, is special for porous media. These processes are simultaneous and coupled. The interplay of the effects may lead to new systematic studies of porous media in a thermal gradient.

Numerical values for the Soret and thermo-osmotic coefficients were computed with non-equilibrium molecular dynamics simulations. The two fluid components were identical except for their molecular masses, which had a ratio of 10:1. Two porous systems were used, one of them was in contact with a bulk two-component fluid reservoir (System A), while the other filled the entire system volume (System B). Both systems were closed in the sense that the number of particles was constant during the simulations.

We found that both the Soret and thermo-osmotic coefficients depend strongly on the porosity and to some extent on the two components’ ability to wet the matrix particles. The Soret coefficient, given by the ratio between the composition profile and the temperature profile, decreases monotonically from the bulk value at 100% porosity down to about 25% of the bulk value down to 30% porosity. The values at porosities above 80% are in good agreement with previous results for bulk liquids. Below 80%, systems A and B gave quite different values for the Soret coefficient, depending on where fluid samples were taken for compositional analysis. If the compositions were based on samples from the reservoirs in System A, the values were biased by the capacity of the reservoirs to sustain absorption in the matrix. With System B, the Soret coefficient was determined from the mole-fraction and temperature gradients in the matrix. We also found that the preferential absorption of the two components had a dramatic effect on the Soret coefficient in Series A, but not in Series B.

The thermo-osmotic coefficient, given as the ratio between the pressure difference and the temperature difference in the two reservoirs in System B, is 0 at 100% porosity and increases non-monotonically with decreasing porosity with a dip around 40% porosity. The fluid density and mole fraction in the pores show an anomaly in the same porosity region. We have not been able to find the physical reason for this dip other than it may be related to the structure of the matrix. This illustrates that the absorption and perhaps also the permeability of the porous medium is a complex function of porosity due to the geometry of the matrix structure versus the size of the particles. In the present case, the effect of the Soret equilibrium on the thermo-osmotic coefficient is small and vice versa. The thermo-osmotic coefficients are surprisingly large, and of the same order of magnitude as has been observed in frost heave.

We show that a thermodiffusion coefficient may be defined for porous systems by analogy to the definition for bulk fluids. If the porous medium is non-selective with respect to the fluid components, this definition gives the Soret coefficient by direct analogy to bulk fluids.

Our findings have two important implications for experimental designs made to measure the Soret coefficient in porous media. (1) When the Soret coefficient is measured with the packed column method and fluid samples are drawn from outside the porous medium, the values may be distorted by absorption in the medium. (2) The Soret effect may depend on variations in the wettability.

## Data Availability

The computer code used for the simulations is available on request.

## Appendix A: Definitions of the coefficients in Eqs. (14)–(16)

The transformation from Eqs. (2)–(3) to (10)–(12) gives the following new coefficients:

A1 $$\begin{aligned} L_{VV}&= L_{11} V_1^2 + L_{12} V_2 V_1 + L_{21} V_1 V_2 + L_{22} V_2^2 = L_{11} V_1^2 \nonumber \\&\quad + 2L_{12} V_1 V_2 + L_{22} V_2^2 \end{aligned}$$

A2 $$\begin{aligned} L_{DD}&= \frac{L_{11}}{x_1^2} + \frac{L_{22}}{x_2^2} - \left( \frac{L_{12}}{x_1 x_2} + \frac{L_{21}}{x_2 x_1} \right) \nonumber \\&= \frac{L_{11}}{x_1^2} + \frac{L_{22}}{x_2^2} - 2\frac{L_{12}}{x_1 x_2} \end{aligned}$$

A3 $$\begin{aligned} L_{qV}&= L_{q1} V_1 + L_{q2} V_2 = L_{Vq} \end{aligned}$$

A4 $$\begin{aligned} L_{qD}&= \frac{L_{q1}}{x_1} - \frac{L_{q2}}{x_2} = L_{Dq} \end{aligned}$$

A5 $$\begin{aligned} L_{VD}&= \left( \frac{L_{11}}{x_1} - \frac{L_{12}}{x_2} \right) V_1 + \left( \frac{L_{21}}{x_1} - \frac{L_{22}}{x_2} \right) V_2 = L_{DV} \end{aligned}$$

The coefficient $L_{qq}$ is unaffected, and the symmetry of the *L*-coefficients ($L_{ij}=L_{ji}$) is preserved by the transformation.

## Appendix B: Definitions of reduced quantities in Lennard–Jones units

**Table:**

Property	Dimension	Definition in L-J units
Temperature	K	$T^* = \frac{k_\text {B} T}{\varepsilon _{11}}$
Pressure	Pa	$P^* = P \frac{\sigma _{11}^3}{\varepsilon _{11}}$
Number density	mole $\text {m}^{-3}$	$\rho ^* = \rho \sigma _{11}^3$
Heat flux	Joule $\text {m}^{-2}\text {s}^{-1}$	$J_q^* = J_q \frac{\sigma _{11}^3}{\varepsilon _{11}} \left( \frac{m_1}{\varepsilon _{11}} \right) ^{1/2}$
Molar (particle) flux	mole $\text {m}^{-2}\text {s}^{-1}$	$J_i^* = J_i \sigma _{11}^3 \left( \frac{m_1}{\varepsilon _{11}} \right) ^{1/2}$
Diffusion flux	mole $\text {m}^{-2}\text {s}^{-1}$	$J_D^* = J_D \sigma _{11}^3 \left( \frac{m_1}{\varepsilon _{11}} \right) ^{1/2}$

**Table:**

Volume flux	m $\text {s}^{-1}$	$J_V^* = J_V \sigma _{11}^6 \left( \frac{m_1}{\varepsilon _{11}} \right) ^{1/2}$
Soret coefficient	$\text {K}^{-1}$	$S^* = S \frac{\varepsilon _{11}}{k_\text {B}}$
Thermo-osmotic coefficient	Pa $\text {K}^{-1}$	$D_\mathrm{P}^* = D_\mathrm{P} \frac{\sigma _{11}^3}{k_\text {B}}$
Heat of transfer (molar)	Joule $\text {mole}^{-1}$	$\left( q_1^* \right) ^* = q_1^* \frac{1}{\varepsilon _{11}}$
Heat of transfer (diffusion)	Joule $\text {mole}^{-1}$	$\left( q_D^* \right) ^* = q_D^* \frac{1}{\varepsilon _{11}}$
Heat of transfer (volume)	Joule $\text {m}^{-3}$	$\left( q_V^* \right) ^* = q_V^* \frac{1}{\varepsilon _{11}\sigma _{11}^3}$
